# Screening of cacna1a and ATP1A2 genes in hemiplegic migraine: clinical, genetic and functional studies

**DOI:** 10.1186/1129-2377-14-S1-P26

**Published:** 2013-02-21

**Authors:** C Sintas, O Carreño, R Corominas, SA Serra, M Vila, N Fernández-Castillo, C Toma, R Pons, M Llaneza, MJ Sobrido, D Grinberg, MA Valverde, JM Fernández-Fernández, A Macaya, B Cormand

**Affiliations:** 1 Departament de Genètica, Facultat de Biologia, Universitat de Barcelona, Spain; 2 Department of Experimental and Health Sciences, UPF, Spain; 3 Pediatric Neurology Research Group, Vall d’Hebron, Spain; 4 First Department of Pediatrics, Agia Sofia Hospital, University of Athens, Athens, Greece; 5 Sección de Neurología, Complejo Hospitalario Arquitecto Marcide-Novoa Santos, Ferrol, Spain; 6 Fundación Pública Galega de Medicina Xenómica, Santiago de Compostela, Spain; 7 Laboratory of Molecular Physiology and Channelopathies, Department of Experimental and Health Sciences, UPF, Spain; 8 Pediatric Neurology Research Group, Vall d’Hebron Research Institute, Spain

## Introduction

Hemiplegic migraine (HM) is a rare and severe subtype of autosomal dominant migraine, characterized by a complex aura including some degree of motor weakness. Mutations in three genes (CACNA1A, ATP1A2 and SCN1A) have been detected in familial and in sporadic cases. This genetically and clinically heterogeneous disorder is often accompanied by permanent ataxia, epileptic seizures, mental retardation, and chronic progressive cerebellar atrophy.

## Objectives

To perform an exhaustive mutational screening of the CACNA1A and ATP1A2 genes in 18 HM patients.

## Methods

Direct sequencing of PCR amplicons, Multiplex Ligation-dependent Probe Amplification (MLPA), Quantitative Multiplex PCR of Short Fluorescent fragments (QMPSF), heterologous expression and electrophysiology, ouabain survival assay.

## Results

We identified four previously described missense CACNA1A mutations (p.Ser218Leu, p.Thr501Met, p.Arg583Gln and p.Thr666Met) and two missense changes in the ATP1A2 gene, the previously described p.Ala606Thr and the novel variant p.Glu825Lys. Additionally, a quantitative analysis was performed to detect exonic duplications or deletions in the CACNA1A gene using MLPA and QMPSF, with negative results. Functional studies were performed for the CACNA1A p.Thr501Met mutation and the ATP1A2 p.Glu825Lys change, the first having been previously described only in association with the EA2 phenotype.

## Conclusion

This genetic screening allowed the identification of more than 30% of the disease alleles. Functional studies performed with two of the identified changes suggest that they are disease-causing.
